# Autophagy is involved in oligodendroglial precursor-mediated clearance of amyloid peptide

**DOI:** 10.1186/1750-1326-8-27

**Published:** 2013-08-10

**Authors:** Wenxia Li, Yifen Tang, Zhiqin Fan, Ya Meng, Guang Yang, Jia Luo, Zun-Ji Ke

**Affiliations:** 1 Key Laboratory of Nutrition and Metabolism, Institute for Nutritional Sciences, Shanghai Institutes for Biological Sciences, Chinese Academy of Sciences, Graduate School of the Chinese Academy of Sciences, Shanghai 200031, China; 2 Department of Internal Medicine, University of Kentucky College of Medicine, Lexington, KY 40536, USA; 3 Shanghai Clinical Center, CAS / Shanghai Xuhui Central Hospital, Shanghai, China

**Keywords:** Alzheimer’s disease, β-amyloid degradation, Autophagy, Endocytosis, NG2 cells

## Abstract

**Background:**

Accumulation of β-amyloid peptides is an important hallmark of Alzheimer’s disease (AD). Tremendous efforts have been directed to elucidate the mechanisms of β-amyloid peptides degradation and develop strategies to remove β-amyloid accumulation. In this study, we demonstrated that a subpopulation of oligodendroglial precursor cells, also called NG2 cells, were a new cell type that can clear β-amyloid peptides in the AD transgene mice and in NG2 cell line.

**Results:**

NG2 cells were recruited and clustered around the amyloid plaque in the APPswe/PS1dE9 mice, which is Alzheimer’s disease mouse model. In vitro, NG2 cell line and primary NG2 cells engulfed β-amyloid peptides through the mechanisms of endocytosis in a time dependent manner. Endocytosis is divided into pinocytosis and phagocytosis. Aβ_42_ internalization by NG2 cells was mediated by actin-dependent macropinocytosis. The presence of β-amyloid peptides stimulated the autophagic pathway in NG2 cells. Once inside the cells, the β-amyloid peptides in NG2 cells were transported to lysosomes and degraded by autophagy.

**Conclusions:**

Our findings suggest that NG2 cells are a new cell type that can clear β-amyloid peptides through endocytosis and autophagy.

## Background

The major neuropathological hallmarks of Alzheimer’s disease (AD) are selective loss of neurons and the formation of amyloid plaques and neurofibrillary tangles [1]. It is suggested that accumulation of β-amyloid peptides (Aβ) plays a central role in pathophysiological procedure of AD [2,3]. Cellular engulfment of Aβ is an important mechanism for clearing the harmful protein in the brain; microglia, astrocyte and neuron are known cell types capable of clearing Aβ through various putative receptors and transporters [4]. Once internalized, Aβ can be degraded by various proteases. Autophagy is an important cellular self-regulatory process involving protein degradation and recycling. It is essential in maintaining neuronal homeostasis, and its dysfunction has been directly linked to a number of neurodegenerative disorders [5,6]. Recent finding suggests that Aβ may be degraded by autophagy/lysosome pathway [7].

In the brain, a subpopulation of glia termed oligodendroglial precursor cells (OPCs). These cells express NG2 (a chondroitin sulfate proteoglycan), are therefore called NG2 cells [8,9], they are distinct from astrocytes, microglia, mature oligodendrocytes and neurons [10]. NG2 cells are abundant in adult brain and comprise 5–8% of brain cells [11]. Morphologically, NG2 cells have small cell bodies and multiple branched processes. In grey matter, these processes tend to have a radial orientation, whereas in white matter, the processes are more longitudinal and aligned with the nerve fibers. These fine cellular processes also ensheath synaptic profiles [12]. Neurons also have synaptic junctions with NG2 cells [13,14]. It is suggested that NG2 cells are a widely distributed stem-like cells in the adult brain. Usually, NG2 cells are induced to differentiate to glial cells, but under the appropriate circumstances, they might generate neurons [15,16]. More importantly, NG2 cells become rapidly activated in response to a variety of CNS insults, including physical trauma [17], excitotoxic lesions [18], viral infection [19], and exposure to chemicals [20].

In this study, we examined the role of NG2 cells in Aβ_42_ clearance in mice. We demonstrated that the number of active NG2 cells was increased and the cells were clustered around the amyloid plaque. In addition, cultured NG2 cells were able to uptake and clear Aβ_42_. Upon internalization most of the Aβ_42_ is transported to lysosomes and degraded by autophagy-lysosome pathway. Our results indicate that NG2 cells can reduce Aβ_42_ through endocytosis and degrade Aβ_42_ by autophagy-lysosome pathway.

## Results

### NG2 cells clustered around the amyloid plaque

**Figure 1:**
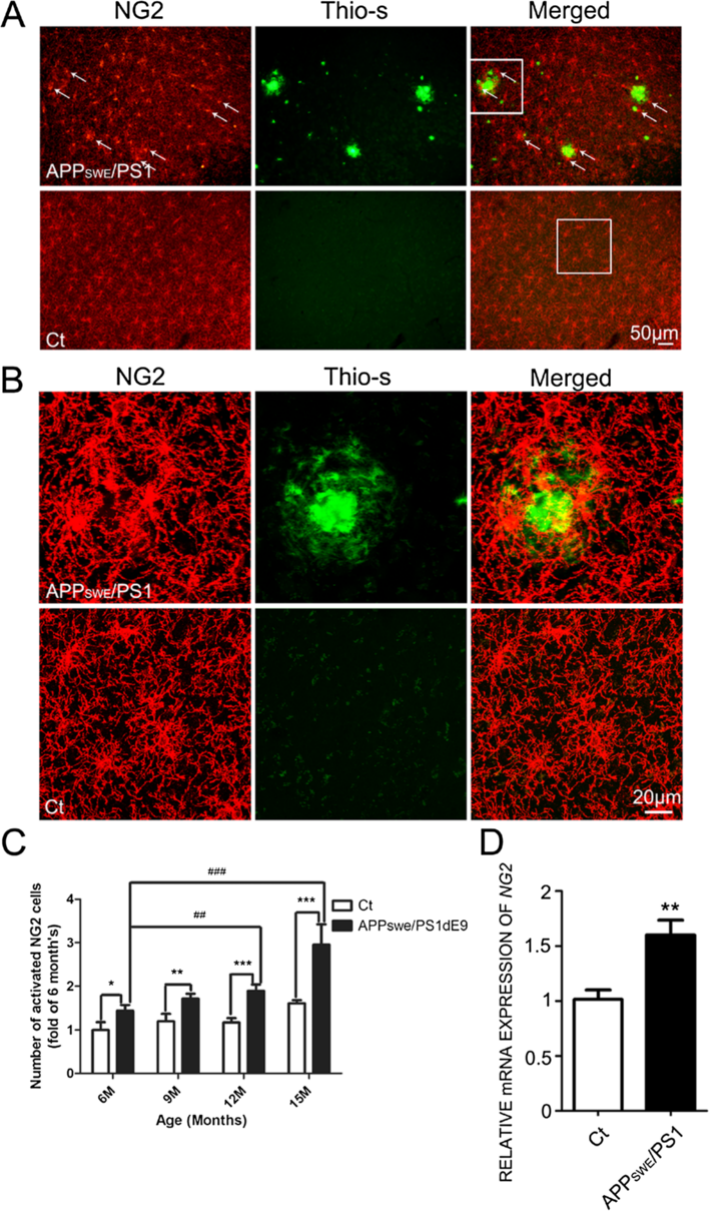
**The presence of NG2 cells adjacent to the amyloid plaque. A.** The presence of NG2 cells in the cortex of 14-month-old APPswe/PS1 mice (top) and control mice (bottom) was demonstrated by immunohistochemistry (IHC) using an anti-NG2 antibody. The amyloid plaque was demonstrated by thioflavin-S staining. Scale bars = 50 μm. **B.** A specified area in panel **A** is shown with higher magnifications. Scale bars = 20 μm. **C.** Activated NG2 cells in the hippocampus of APPswe/PS1 mice (6-15-month-old) and age matched control mice were quantified as described under the Materials and Methods. The results were presented as mean ± SD of five animals. **p < 0.01, ***p < 0.001, compared to age matched control mice. ##p < 0.01, ###p < 0.001, compared to 6-month-old APPswe/PS1 mice. **D**: The expression of NG2 mRNA in 12-month-old mice was determined by real time PCR as described under the Materials and Methods. The results were presented as mean ± SEM of five animals. **p < 0.01, compared to age matched control mice.

It has been demonstrated that the number of NG2 cells as well as the expression of NG2 molecules increases and the cells become hypertrophic surrounding the damage sites in various brain injury models [18,20-23]. We examined the localization and morphology of the NG2 cells in the APPswe/PS1dE9 mice, which express familial AD-causing mutated forms of human APP (APPswe, Swedish familial AD-causing mutation) and presenilin1 (PS1dE9). NG2-positive cells became hypertrophic in the cortex of APPswe/PS1dE9 mice (Figure 1A) and clustered around the amyloid plaques (Figure 1B) in 14-month-old APPswe/PS1 mice. The number of activated NG2 cells increased around 2 fold in 15-month-old APPswe/PS1 mice when compared with control mice (Figure 1C). In addition, the expression of NG2 mRNA increased more than 1.5 fold in 12-month-old APPswe/PS1dE9 mice compared to age matched wild type mice (Figure 1D).

### Engulfment of Aβ_42_ by NG2 cells

**Figure 2:**
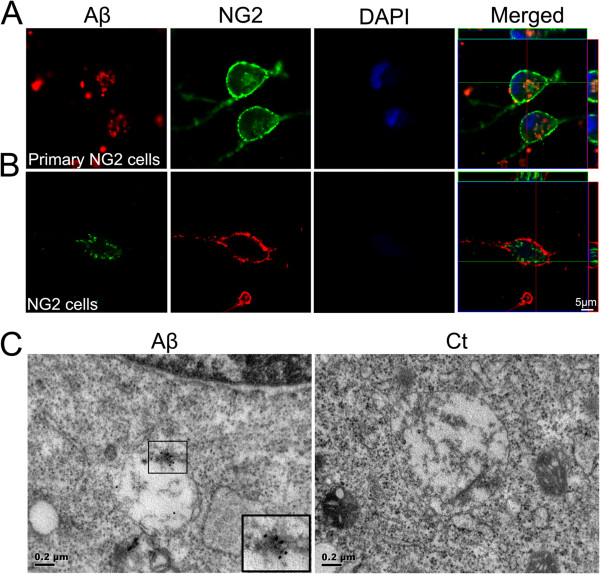
**Aβ**_**42 **_**uptake by NG2 cells.** Primary NG2 cells **(A)** and NG2 cell line **(B)** were plated on coverslips for 18 hours and then incubated with HiLyte Fluor™ 555/488-labeled Aβ_42_ (2 μM) for 24 hours. Cells were then fixed and stained with anti-NG2 antibody. Cell nuclei were visualized by DAPI staining (blue). Aβ_42_ was engulfed by primary cultured NG2 cells and NG2 cells. Scale bars = 5 μm. **C.** Aβ_42_ uptake by NG2 cell line was shown by transmission electron microscopy (TEM). NG2 cell line were exposed to Aβ_42_ for 6 hours, stained with anti-Aβ_42_ IgG antibody followed by donkey anti-rabbit antibody conjugated to colloidal gold (18 nm particle), then processed by TEM. Internalized Aβ_42_ was demonstrated. The inset is an image of internalized Aβ_42_ with higher magnification. Scale bars = 0.2 μm.

**Figure 3:**
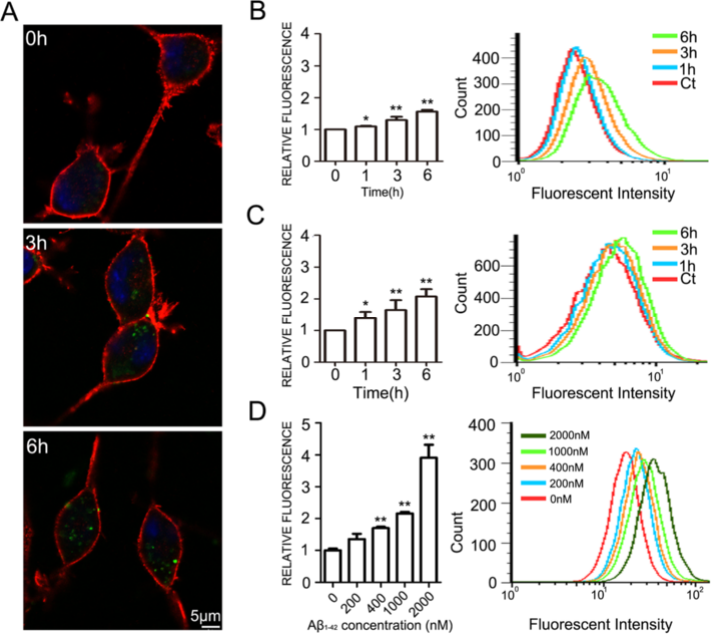
**The time sequence of engulfment of Aβ**_**42 **_**by NG2 cells. A.** NG2 cell line was incubated with HiLyte Fluor™ 488-labeled Aβ_42_ (400 nM) for the indicated times, and stained with anti-NG2 antibody. Aβ_42_ appeared inside the cells after 6 hours of Aβ_42_ incubation. Scale bars = 5 μm. **B-C.** Aβ_42_ engulfed by NG2 cells was quantified with flow cytometry. NG2 cell line **(B)** and primary NG2 cells **(C)** were incubated with HiLyte Fluor™ 488-labeled Aβ_42_ (400 nM) for the indicated times. The amount of Aβ_42_ inside cells were quantified by measuring the intensity of fluoresce with flow cytometry. Data were presented as mean ± SD; *p<0.05, **p<0.01, compared with cells treated with Aβ_42_ for 0 hours. The experiments were replicated three times. **D.** The engulfment of Aβ_42_ by NG2 cells was concentration-dependent. NG2 cell line was incubated with HiLyte Fluor™ 488-labeled Aβ_42_ for the indicated concentrations. The amount of Aβ_42_ inside cells were quantified by measuring the intensity of fluoresce with flow cytometry. Data were presented as mean ± SD; **p<0.01, compared with cells treated without Aβ_42_. The experiments were replicated three times.

Microglia and astrocytes are activated and cluster around amyloid plaques in the brain of AD patients, and both of the cells, especially microglia, play an important role in clearing Aβ [24-27]. The Aβ_42_ variant is more hydrophobic and more prone to fibril formation than Aβ_40_ and it is this longer form that is also the predominant isoform found in cerebral plaques [4]. Aβ_42_ was used in our all experiment. To determine whether NG2 cells were able to engulf Aβ_42_, we incubated the primary NG2 cells with fluorescence-labeled Aβ_42_. The fluorescence-labeled Aβ_42_ was visualized within NG2 cells after 24 hours (Figure 2A). Like primary NG2 cells, NG2 cell line was also able to engulf Aβ_42_ (Figure 2B). The electron microscopy further confirmed that the Aβ_42_ was distributed in the cytosol of NG2 cells (Figure 2C). The density of fluorescence-labeled Aβ_42_ initially increased after incubation for one hour, and levels further increased as incubation time increased (Figure 3). Furthermore, the engulfment of Aβ_42_ by NG2 cells was concentration-dependent (Figure 3D).

### Actin is involved in the engulfment of Aβ_42_

**Figure 4:**
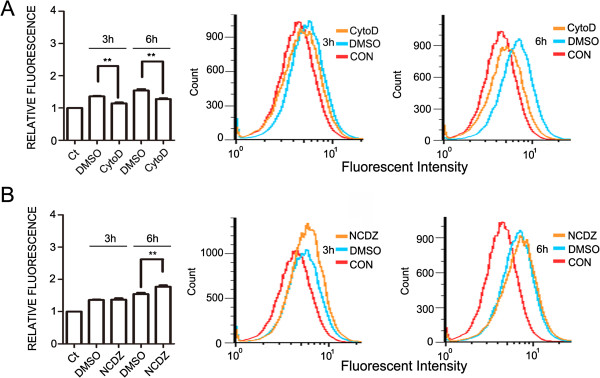
**Effect of cytochalasin D and nocodazole on Aβ**_**42 **_**uptake by NG2 cells.** NG2 cell line were treated with cytochalasin D **(A)**, an inhibitor of actin polymerization or nocodazole **(B)**, an agent that disrupts microtubule dynamics for 30 minutes prior to the addition of HiLyte Fluor™ 488-labeled Aβ_42_ (400 nM) for the indicated times. The amount of Aβ_42_ inside cells were quantified by measuring the intensity of fluoresce with flow cytometry. Data were presented as mean ± SD; **p<0.01, compared with cells without any treatment (Ct). The experiments were replicated three times.

The phagocytosis and pinocytosis are two major forms for cells to uptake extracellular substances. Microglia can engulf Aβ by macropinocytosis [27]. To determine the mechanism for the engulfment of Aβ_42_ by NG2 cells, we treated the NG2 cell line with nocodazole that causes depolymerization of microtubules and cytochalasin D that inhibits actin polymerization. The cytochalasin D reduced the engulfment of Aβ_42_ measured by flow cytometry (Figure 4A). Nocodazole had little effect on Aβ_42_ internalization after 3 hours of exposure but modestly increased Aβ_42_ content following 6 hours of exposure (Figure 4B).

### The Aβ_42_ was degraded by lysosome pathway

**Figure 5:**
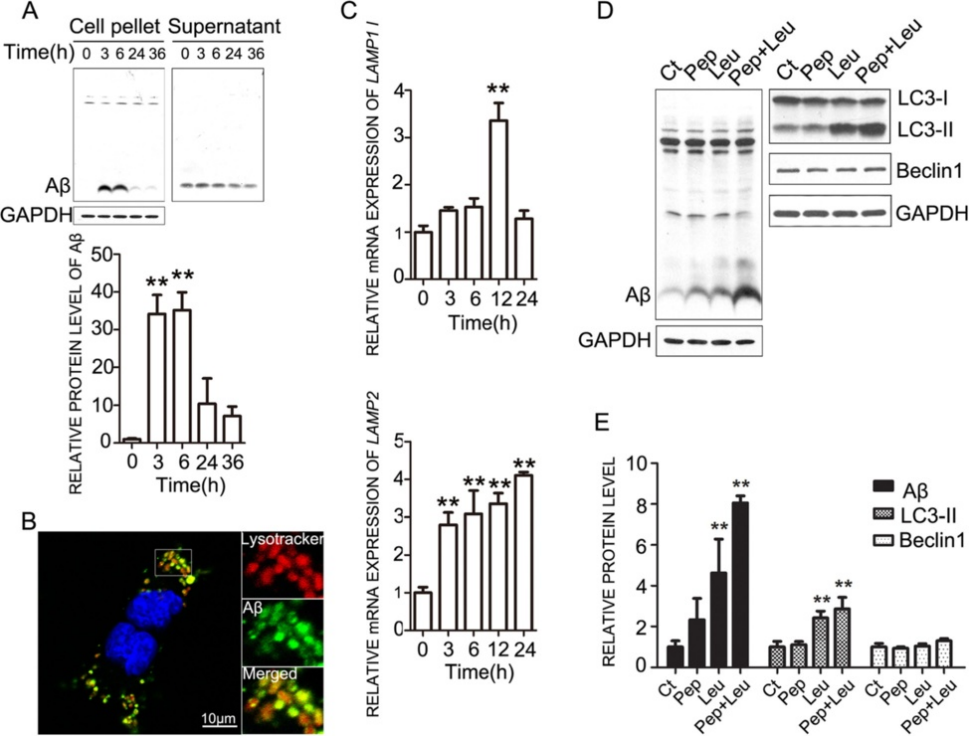
**Localization of Aβ**_**42 **_**in lysosomes. A.** Aβ_42_ in cellular fraction in NG2 cell line and culture supernatant was measured by immunoblots. The results were quantified and presented as mean ± SD; **p<0.01, denote significant difference from that at 0 hours. The experiment was replicated three times. **B.** NG2 cell line was incubated with HiLyte Fluor™ 488-labeled Aβ_42_ (400 nM) for 24 hours, then treated with lysotracker (40 nM) for 30 minutes. Cell nuclei were labeled with DAPI. Aβ_42_ was co-localized with the signal of lysotracker. Scale bars = 10 μm. **C.** The mRNA expression of *LAMP1* and *LAPM2* in NG2 cell line was measured by RT-PCR after treatment with Aβ_42_ at the indicated times. Data were presented as mean ± SD; **p<0.01, compared with cells treated with Aβ_42_ for 0 hours. The experiments were replicated three times. **D and E.** NG2 cell line was exposed to Aβ_42_ for 6 hours, then treated with leupeptin (20 μM) and pepstatin A (20 μM) for 18 hours. The levels of cellular Aβ_42_, and LC3, and beclin1 proteins were determined by immunoblotting and normalized to the expression of GAPDH. Data were presented as mean ± SD; *p<0.05, **p<0.01, denote significant difference from the group treated with Aβ_42_ alone (Ct). The experiments were replicated three times.

To determine the fate of Aβ_42_ after its internalization, we examined the Aβ_42_ content in cellular fraction in NG2 cell line and cell-culture supernatant. Aβ_42_ in the cellular fraction increased within 3 hours of Aβ_42_ incubation then decreased from 24 hours, indicating that Aβ_42_ was first internalized and then degraded (Figure 5A). The Aβ_42_ in the culture supernatant decreased over time, supporting that Aβ_42_ was taken up by the cells. To determine whether lysosome pathway is involved in Aβ_42_ degradation, we investigated the intracellular distribution of Aβ_42_ after its internalization. NG2 cell line was exposed to HiLyte Fluor™-488-labeled Aβ_42_ and lysotracker dye for visualizing lysosomes. As shown in Figure 5B, Aβ_42_ is localized in lysosomes. Aβ_42_ exposure also increased the mRNA expression of Lysosomal-associated membrane proteins (*LAMP*), *LAMP1* and *LAMP2* (Figure 5C). Leupeptin and pepstatin, the inhibitors for major cysteine and aspartyl proteases for lysosomal proteolysis, blocked the degradation of Aβ_42_ in the NG2 cells (Figure 5D). These data suggested that internalized Aβ_42_ was transported into the lysosome and degraded by the lysosome-dependent pathway.

### Autophagy involved in the degradation of Aβ_42_ in NG2 cells

**Figure 6:**
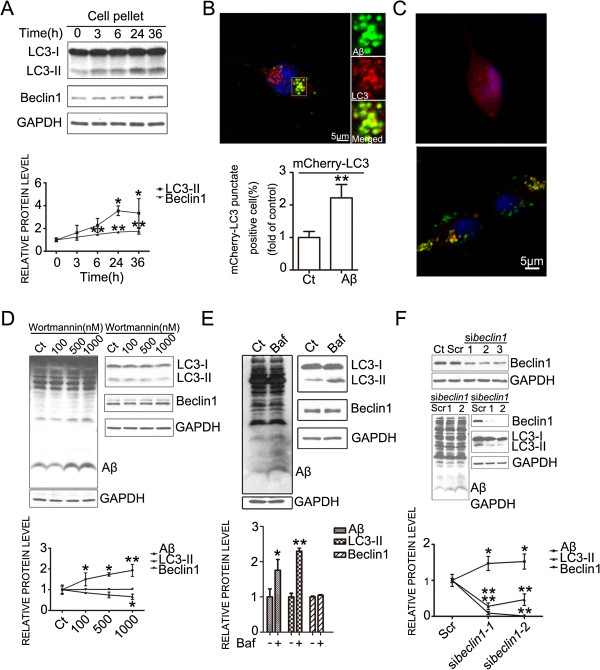
**Autophagy was involved in the degradation of Aβ**_**42**_**. A.** NG2 cell line was treated with Aβ_42_ for specified times. The expression of LC3-II, and beclin1 was determined by immunoblotting. **B.** NG2 cell line was incubated with HiLyte Fluor™ 488-labeled Aβ_42_ (400 nM) for 24 hours, and immunostained using an anti-LC3-II antibody. Cell nuclei were labeled with DAPI. Scale bars = 5 μm. **C.** NG2 cell line was transiently transfected with a mCherry–LC3 plasmid, and autophagosomes were demonstrated by mCherry–LC3 puncta. Scale bars = 5 μm. **D*.*** NG2 cell line was exposed to Aβ_42_ for 6 hours, and then treated with wortmannin at the indicated concentrations for 18 hours. Aβ_42_, and the expression of LC3, and beclin1 was determined by immunoblotting (top panel). The results were quantified (bottom panel). **E*.*** NG2 cell line was exposed to Aβ_42_ for 6 hours, then treated with bafilomycin A1 (0.2 nM) for 18 hours. Aβ_42_, the expression of LC3, and beclin1 was determined by immunoblotting (top panel). The results were quantified (bottom panel). **F.** Top panel**:** NG2 cell line treated with specific *beclin1* siRNA oligonucleotides (si*Beclin1*-1, si*Beclin1*-2, si*Beclin1*-3), scramble RNA oligonucleotides for 24 hours. The expression of beclin1 was analyzed by immunoblotting. Ct: no treatment; Scr: scramble RNA oligonucleotides. Middle panel: NG2 cell line was transfected with *beclin1* siRNA for 24 hours, then treated with Aβ_42_ for another 24 hours. Aβ_42_, the expression of LC3, and beclin1 was determined by immunoblotting. Bottom panel: Results were quantified. All data were presented as mean ± SD; *p<0.05, **p<0.01, denote significant difference. All the experiments were replicated three times.

Since autophagy is involved in amyloid-beta peptide metabolism and clearance [7,28-30], we tested whether autophagy regulated the degradation of Aβ_42_ in NG2 cells. Aβ_42_ treatment increased the expression of autophagy related proteins, including LC3 and beclin1 (Figure 6A). Furthermore, Aβ_42_ was co-localized with LC3, suggesting that they were enclosed by autophagosomes (Figure 6B). Aβ_42_ treatment increased the number of mCherry–LC3 puncta–positive cells, suggesting an induction of autophagy (Figure 6C). Autophagy inhibitors, wortmannin (Figure 6D) and bafilomycin A1 (Figure 6E), decreased the Aβ_42_ degradation. The expression of LC3-II decreased when treated with wortmannin (Figure 6D) and increased when treated with bafilomycin A1 (Figure 6E). Beclin1 did not show changes when treated with wortmannin (Figure 6D) or when treated with bafilomycin A1 (Figure 6E) in NG2 cells. Knockdown of *beclin1* in NG2 cells using a *beclin1* specific siRNA also increased the accumulation of Aβ_42_ (Figure 6F). Together, these results suggest that Aβ_42_ could induce autophagy which directed Aβ_42_ to lysosome-dependent protein degradation in NG2 cells.

## Discussion

Amyloid plaques, which consist of aggregates of Aβ in the brain, are the predominant pathological change in AD patients. The overload of Aβ combined with hyperphosphorylated neurofibrillary tangles (NFTs), neuronal loss, inflammation, and oxidative stress make synaptic dysfunction and behavioral changes [31,32]. It is important to understand the mechanisms underlying the clearance of the amyloid protein in the brain. The levels of Aβ peptides within the brain are tightly regulated by mechanisms controlling their generation and clearance [3]. In human CNS, the rate for production and clearance of Aβ is 7.6% per hour and 8.3% per hour, respectively; therefore there is no accumulation and deposition of Aβ in the normal brain [33]. However, even a modest perturbation in Aβ clearance will result in an imbalance between production and clearance, and cause an accumulation of Aβ peptides within the brain and their subsequent deposition into plaques. The excessive accumulation of Aβ may trigger the onset of AD, as suggested by the “amyloid hypothesis” [2]. Activated microglial cells and astrocytes are associated with amyloid plaques [24,25,27,34,35]. Microglia and astrocytes are shown to engulf and degrade Aβ in vitro and in situ [24,25,27,34]. In this study, we demonstrate that NG2 cells are recruited and clustered in the area adjacent to amyloid plaques. The number of active NG2 cells is increased and NG2 mRNA is upregulated. NG2 cells are able to internalize and degrade Aβ_42_ and a new cell type to clear Aβ.

### Mechanisms of NG2 cell-mediated Aβ_42_ uptake

Extracellular substances can be taken up by cells and transported to intracellular compartments through two major pathways, the phagocytosis and the pinocytosis. Phagocytosis is restricted to specialized phagocytic cells and is responsible for receptor-dependent uptake of large particles such as bacteria. Pinocytosis encompasses several distinct mechanisms [36,37]. Macropinocytosis belongs to dynamin-independent pinocytosis, which can be defined as a transient, growth factor-induced, actin-dependent endocytic process that leads to internalization of fluid and membrane into large vacuoles [38]. Macropinosome formation is an actin based process. Vacuole formation is probably the result of local actin cortex destabilization [37]. A recent study suggests that microglia internalize Aβ through fluid phase macropinocytosis [27]. Cytochalasin D, the inhibitor of actin polymerization, inhibited the engulfment of Aβ_42_ by NG2 cells, and nocodazole, the inhibitor of tubulin polymerization had little effect on Aβ_42_ engulfment. Microtubules have diverse roles in the cellular function, including vesicular transportation that facilitates Aβ_42_ degradation. They play a role in late steps of endocytosis and are involved in the traffic between early and late compartments [39]. Translocation of endosomes and lysosomes occurs along microtubules and is independent of the intermediate filament and microfilament networks [40,41]. When the microtubules are depolymerized with nocodazole, translocation of endosomes and lysosomes is inhibited, which will affect the transportation of Aβ_42_ for its degradation in lysosome, resulting in accumulation of Aβ_42_ in cytoplasm. Our data did show nocodazole modestly increased Aβ_42_ amount in NG2 cells. These results suggest that Aβ_42_ internalization by NG2 cells may be mediated by actin-dependent macropinocytosis and the microtubule-dependent process may be involved in its degradation.

The accumulation of Aβ results from the impairment of balance between producing and clearance of Aβ. So far, activated microglia and astrocytes are known cell types that can engulf Aβ. Apart from activated microglia and astrocytes, neurons can internalize Aβ peptide. Our results show that both primary NG2 cells and NG2 cell lines are able to engulf and clear Aβ_42_, adding a new cell type to clear Aβ in CNS. The time sequence of NG2 cell uptake of Aβ_42_ is similar to that of microglia [27]. Based on our observation the ability of NG2 cells to clear Aβ is much weaker than microglia. Therefore, in AD brain, microglia is the major cell type to clear Aβ. Microglia expresses a number of putative Aβ transporters, such as scavenger receptor for advanced glycation end products (RAGE) [42], formyl peptide receptor-like 1 (FPRL1) [43,44] and toll-like receptors (TLRs) [45]. These transporters may facilitate microglia-induced clearance of Aβ. Mandrekar et al. (2009) also demonstrate that microglia uptake of Aβ is mediated by fluid phase macropinocytosis both in vitro and in vivo, and tubulin depolymerization and actin polymerization are required for the process [27]. Our data support that NG2 cell-mediated Aβ_42_ uptake depends on actin polymerization but not tubulin depolymerization.

Cytochalasin D does not completely inhibit Aβ_42_ internalization in NG2 cells, suggesting that there may be other pathways that participate in Aβ_42_ internalization. Aβ can bind to various membrane biomolecules, including lipids, proteins and proteoglycans. A number of putative Aβ transporters have been identified, such as α7 nicotinic acetylcholine receptor (α7nAChR) [46], apolipoprotein E (ApoE) receptors [47], members of the low-density lipoprotein receptor (LDLR) family [48], scavenger receptor for advanced glycation end products (RAGE) [49], formyl peptide receptor-like 1 (FPRL1) [43,44] and toll-like receptors (TLRs) [4]. It is likely that Aβ also can be internalized through these receptors or transporters.

### Mechanisms of NG2 cell-mediated Aβ_42_ degradation

Recent studies suggest that autophagy/lysosome pathway (ALP) is an important and perhaps compensatory mechanism for intracellular protein degradation [50,51]. The accumulation of lysosomes and their hydrolases within neurons is a well-established neuropathologic feature of AD. The endosomal-lysosomal system is reported to be activated in vulnerable neurons in AD brains [52,53]. It has been suggested that the autophagy/lysosome pathway is involved in the degradation of Aβ [28]. Autophagosomes and other prelysosomal autophagic vacuoles were abundant in AD brains. The transport of autophagic vacuoles and their maturation to lysosomes is impaired, resulting in the accumulations of immature autophagic vacuoles and the inhibition of Aβ clearance [54]. We showed that Aβ_42_ was localized to lysosomes after internalization and the expression levels of lysosomal-associated membrane protein 1 and 2 genes were increased. Moreover, the degradation of Aβ_42_ was inhibited by lysosomal proteolysis inhibitors leupeptin and pepstatin A. The effects of leupeptin supports the role of cysteine protease in degrading Aβ has been documented in earlier studies [55,56]. Pepstatin A increased Aβ_42_ levels without altering LC3-II or beclin1 levels. This may be due to the relative insensitivity of NG2 cells to pepstatin A and a higher concentration may be necessary. Pepstatin A is an inhibitor of aspartic proteinases such as pepsin, cathepsins D and E. The optimal concentrations for pepstatin A are different among cell types. Pepstatin A in combination with Leupeptin did induce more LC3-II accumulation. These results indicated that Aβ_42_ was degraded through the lysosomal pathway.

Protein degradation by the autophagy/lysosomal pathway was initiated by the formation of a double-membrane-limited autophagosome, containing undigested cytoplasmic materials. LC3 serves as a specific marker for autophagy in mammalian cells. It has cytoplasmic form (LC3-I) and autophagosome membrane associated form LC3-II. LC3 I can be transferred to phophatidylethanolamine (PE) in the double membrane of the autophagosome and become a lipidated form of LC3 (LC3-II). The ratio of LC3-II to LC3-I is commonly used as a marker of autophagosome formation and autophagy activation. Digestion of sequestered material within autophagosomes is initiated when lysosomes fuse with the outer membrane of the autophagosome, forming autolysosome/autophagolysosome [30]. Beclin1 is a downstream effecter in the autophagy process and is involved in the recruitment of membranes to form autophagosomes [57-59]. Reduction expression of *beclin1* increases intraneuronal Aβ accumulation, extracellular Aβ deposition, and neurodegeneration in transgenic mice that express human amyloid precursor protein [7]. We demonstrated that Aβ_42_ increased the expression of LC3-II, beclin 1 and LC3 puncta in NG2 cells, indicating that Aβ_42_ could activate autophagy. Furthermore, wortmannin, the chemical inhibitor, and *beclin1* siRNA inhibited autophagy and increased Aβ_42_ accumulation in NG2 cells, indicating that Aβ_42_ degradation was at least partially mediated by the autophagy/lysosomal pathway.

## Conclusions

Amyloid plaques, which consist of aggregates of β-amyloid peptides in the brain, are the predominant pathological change in AD patients. An imbalance between Aβ production and clearance will result in accumulation of Aβ peptides and subsequent deposition into plaques. Autophagy may be a protective response to AD during early pathogenesis and is impaired as the disease progresses. Our studies demonstrated that NG2 cells were clustered around amyloid plaque. NG2 cells engulfed Aβ_42_ through macropinocytosis. The internalized Aβ_42_ was degraded by the autophagy. These findings identified a novel cell type that could participate in the clearance of Aβ_42_ in the brain, which may provide a new insight into the mechanisms of Aβ_42_ degradation. It also potentially offers new strategy for eliminating toxic senile plaques in AD.

## Materials and methods

### Antibodies and reagents

All culture dishes, plates and flasks were obtained from Corning. All chemicals, such as Thioflavine S (T1892), cytochalasin D (C2618), nocodazole (M1404), leupeptin (L9783), pepstatin A (P5318), wortmannin (W3144), bafilomycin A1 (B1793) and platelet-derived growth factor-AA (P3076) were obtained from Sigma-Aldrich unless otherwise mentioned. Fibroblast Growth Factor basic, human Animal-Free recombinant (bFGF, GF003-AF) and Anti-NG2 (rabbit, AB5320) antibodies were obtained from Chemicon. Anti-beclin1 antibody (rabbit, 3495 s) was obtained from Cell Signaling Technology. Anti-6E10 (mouse, sig-39320) antibody was obtained from Signet. Anti- LC3B (rabbit, L7543) was obtained from Sigma-Aldrich. Anti-GAPDH antibody (kc-5G5) was obtained from Kangcheng Bio-tech. HiLyte Fluor™ 555-labeled beta-amyloid (1–42) (60480–01) or HiLyte Fluor™ 488-labeled beta-amyloid (1–42) (60479–01) were obtained from Anaspec. Lysotracker(L7528), trizol (15596018) and anti-Aβ(1–42) antibody(700254) were obtained from Invitrogen. Alex-labeled secondary antibodies were obtained from Molecular Probes. Colloidal gold-affinipure donkey anti-rabbit IgG (18 nm) was obtained from Jackson Laboratories. Agarose II (low gelling temperature biotechnology grade, 0815) was obtained from Amresco.

### Animal

A rodent transgenic animal that expresses familial AD-causing mutated forms of human APP (APPswe, Swedish familial AD-causing mutation) and presenilin1 (PS1△E9) (Jackson Laboratory) were used as an AD model. These animals develop amyloid deposits similar to those found in brains of humans diagnosed with AD [60,61]. Male SD rats at postnatal day 1–2 were obtained from Shanghai SLAC Laboratory Animal Co. Ltd. The procedure for animal surgery was performed in accordance with the Guidelines of Animal Care and Use Committee of the Institute for Nutritional Sciences, Shanghai Institutes for Biological Sciences (SIBS), Chinese Academy of Sciences. Every effort was made to minimize the number of animals used and their suffering.

### Sample collection

For immunohistochemical analysis, animals were anesthetized by i.p. injection of chloral hydrate (500 mg/kg) and were perfused with 10 ml of saline, followed by 100 ml of 4% paraformaldehyde in 0.1 M phosphate buffer (PB, pH 7.2). The brains were removed and post-fixed in the same fixative overnight, and then transferred to 30% sucrose for an additional 24 hrs at 4°C. The brain block was dissected on a Rodent Brain Matrix (ASI Instruments) and sectioned with a sliding microtome (Microm Laborgerate GmbH) at the thickness of 40 μm. For immunoblotting analysis, cultured cells were collected and homogenized in an ice-cold lysis buffer containing 5 mM EDTA, 0.5% NP-40, 0.1% Triton X-100, 0.1% SDS, 10 mg/ml PMSF, 10 μg/ml leupeptin and 100 mM sodium orthovanadate in phosphate buffer saline (PBS). Homogenates were centrifuged at 14,000 revolutions per minute (rpm) for 30 minutes at 4°C and the supernatant fraction was collected. For real-time PCR analysis, animals were anesthetized by i.p. injection of chloral hydrate (500 mg/kg) and were perfused with 10 ml of saline. The brains were removed and cerebral cortex were collected for total RNA extraction.

### Immunohistochemical staining

For immunofluorescence staining, sections were pre-incubated with 0.3% Triton X-100 in PBS for 10 minutes and blocked with 1%BSA, 0.1% Triton X-100 for 1 hour. Then the sections were incubated with anti-NG2 antibody (rabbit, 1:500) at 4°C overnight and the Alexa Fluor 555-conjugated goat anti- rabbit IgG (1:1000, Invitrogen, A-21428) at room temperature for 2 hours. After that, DAPI (1 μg/ml) was added in the washing buffer for 5 minutes. Brain sections were examined with an Olympus BX51 microscope (Olympus America Inc.) or Zeiss LSM 510 Meta confocal microscope (Carl Zeiss microImaging Inc.).

### Thioflavin-S staining

The procedure for thioflavin-S staining has been previously described [62]. Briefly, sections were stained with 0.05% thioflavin-S in 50% ethanol in the dark for 8 minutes, followed by two rinsing in 80% ethanol for 10 seconds each and three washes in large volumes of distilled water. Slides were then incubated in a high concentration of phosphate buffer (411 mM NaCl, 8.1 mM KCl, 30 mM Na_2_HPO_4_, 5.2 mM KH_2_PO_4_, pH 7.2) at 4°C for over 30 minutes. After that slides were briefly rinsed with distilled water, and then sealed with coverslips.

### Quantification of activated NG2 cells number

NG2-positive cells with larger cell body, cell body stained more heavily with antibody against NG2, thicker and shorter processes were considered as activated NG2 cells [19,20]. Only NG2 cells with clearly visible nuclei were counted. The number of activated NG2 cells in the hippocampus was quantified by manually counting as previously described [23,63,64] with some modifications. Five animals of each group were used for the quantifications . For each mouse, the brain was sectioned at the thickness of 40 μm. Sections containing hippocampus were collected from the Bregma level -1.22 mm to -2.3 mm. Every fifth section was collected and used for NG2 staining. Five sections per animal were used for quantifications. Digital images are acquired by a Olympus BX51 microscope system equipped with a DP72 digital camera (Olympus America Inc.) using a 10× objective lens. All parameters were held constant for all sections. The area of hippocampus was measured by ImageJ (1.44p). The result was presented as number of activated NG2 cells per unit area (number of activated NG2 cells/mm^2^).

### Cell cultures

Primary rat oligodendrocyte precursor cells were derived from the brains of SD rat at postnatal day 1–2 as previously described [65] with some modifications. Briefly, cerebral cortices from postnatal day 1–2 SD rats were dissected, minced and digested. Dissociated cells from one rat were plated in two 75 cm^2^ tissue culture flasks coated by 100 μg/ml Poly-D-Lysine(Sigma-Aldrich, p7886). Cell cultures were maintained in Dulbecco’s modified Eagle’s medium (DMEM, Invitrogen, 12100–046) supplemented with 10% fetal bovine serum (Hyclone, SV30087.02), 10% of horse serum (Invitrogen, 26050–088), 100 U/ml streptomycin and 100 U/ml penicillin G (Invitrogen, GB15140-122) at 37°C in humidified air with 5% CO_2_ for 7 to 10 days without changing the culture medium. After that, the flasks were sealed and shaken at 230 rpm at 37°C for 3 hours to remove microglial cells. The medium was removed and replaced with 10 ml DMEM containing 10% fetal bovine serum, 10% of horse serum, 100 U/ml streptomycin and 100 U/ml penicillin G. The flasks were shaken again for 20 hours at 260 rpm at 37°C. The medium containing floating cells was collected and placed in Petri dishes for 30 minutes at 37°C. The non-adherent cells (oligodendrocyte precursor cells) were collected and replated on a Poly-D-Lysine-coated 24-well plate at a density of 5 × 10^4^ cells/well. Cells were maintained in SATO medium containing 1% horse serum, 10 ng/ml platelet-derived growth factor, and 5 ng/ml bFGF. The NG2 cell line was kindly provided by Dr. Jacqueline Trotter (Johannes Gutenberg University of Mainz, Mainz, Germany) and cultured on Poly-D-Lysine coated coverslips in SATO medium containing 1% horse serum [66]. The NG2 cell line was a murine oligodendroglial precursor cell line and generated by immortalization of mitotic oligodendrocyte precursor cells with retroviral vectors containing the t-neu oncogene. The cell line has the properties of oligodendrocyte precursor and can differentiate into myelin-associated glycoprotein (MAG)-positive oligodendrocytes. It is a widely used as oligodendrocyte precursor cells in many in vitro studies [67-69].

### Aβ_42_ preparation

Soluble species of Aβ_42_ was prepared according to the instruction of the manufacturer (Anaspec). Briefly, the lyophilized Aβ_42_ peptide powder was dissolved in 1.0% NH_4_OH (provided by the manufacturer) to get a stock solution. Immediately dilute this stock solution with 1× PBS to a concentration of approximately 1 mg/mL. Gently vortex to mix. Reconstituted peptide was aliquoted into several freezer vials and stored at -80°C.

### Immunocytochemistry

Primary oligodendrocyte precursor cells and NG2 cell line were plated on coverslips in a 24-well plate at a density of 5 × 10^4^ and 3 × 10^4^cells/well, respectively for 18 hours. Cells were incubated with HiLyte Fluor™ 555- or 488-labeled Aβ_42_ (2 μM) for 24 hours, and then fixed in 4% paraformaldehyde. After permeabilization with 0.1% Triton X-100, cells were washed with PBS three times and blocked in a solution containing 3% BSA and 0.1% Triton X-100 in PBS for 1 hour. Cells were incubated with primary anti-NG2 antibody (1:500) at 4°C overnight and then washed three times with PBS, incubated with Alexa488- or 555-conjugated goat anti-rabbit secondary antibodies at a 1:1000 dilution for 1 hour, and then 1 μg/ml DAPI for 5 minutes. Coverslips were mounted on slides and observed using a Zeiss LSM 510 Meta confocal microscope (Carl Zeiss microImaging Inc.). For Aβ_42_’s uptake study, the NG2 cell line were incubated with HiLyte Fluor™ 488-labeled Aβ_42_ (400 nM) for the indicated times. Cells were fixed in 4% paraformaldehyde. After permeabilization with 0.1% Triton X-100, cells were blocked in a solution containing 3% BSA and 0.1% Triton X-100 in PBS for 1 hour. Cells were then processed for NG2 immunocytochemistry as described above. For visualizing lysosomes, the cells were labeled with lysotracker (40 nM, 30 minutes) and then fixed, permeabilized and stained with DAPI.

### Transmission electron microscopy

Immunoelectron microscopy was performed as described previously [70,71]. NG2 cell line treated with Aβ_42_ for 6 hours was collected and fixed with 2.5% glutaraldehyde in 0.1 M phosphate buffered saline (PBS; pH 7.4) at room temperature for 1 hour and washed 3 times with PBS. The cell pellets were then embedded in 2% agarose II and postfixed with 1% Osmium tetroxide at room temperature for 1 hour. The fixed cell pellets were rinsed three times with distilled water and three times with PBS. The pellets were dehydrated through an ethanol (EtOH) dilution series up to 100% EtOH and then infiltrated in propylene oxide/ Epon812 resin mixture. Then the pellets were infiltrated in 100% Epon812 for 1 hour. Subsequently, the pellets were embedded in 100% Eponate resin and sectioned. Ultrathin sections were treated with 1% sodium periodate for 10 minutes. After washed with ddH_2_O, sections were blocked in 2%FBS in PBS without Ca^2+^/Mg^2+^ for 30 minutes and incubated with rabbit anti-Aβ_42_ IgG antibody (1:250) overnight at 4°C followed by donkey anti-rabbit antibody conjugated to colloidal gold (18 nm particle for Aβ_42_ (1:20), Jackson Laboratories) for 2 hours at room temperature. Sections were double-stained with uranyl acetate and lead citrate, and examined under a JEOL JEM1230 electron microscope (JEOL).

### Quantification of Aβ_42_ by flow cytometry

Quantification of Aβ_42_ by flow cytometry was performed as previously described with some modifications [27]. Briefly, primary oligodendrocyte precursor cells and NG2 cell line were plated at a density of 2 × 10^5^ cells/well in a 6-well plate overnight. The cells were incubated with HiLyte Fluor™ 488-Aβ_42_ (400 nM) for the indicated times. For nocodazole and cytochalasin D treatment, NG2 cells were preincubated with 150 nM nocodazole and 5 μg/ml cytochalasin D for 30 minutes and then incubated with HiLyte Fluor™ 488-Aβ_42_ (400 nM) for the indicated times. Cells were removed by the treatment of 0.01% trypsin (Invitrogen, 25200–072), centrifuged at 1,000 g for 5 minutes, and washed with PBS twice. After that, cells were resuspended in PBS for the analysis using a FACScan cytometer (BD Biosciences) equipped with a FITC signal detector FL1 (excitation 488 nm, green).

### Immunoblotting

The procedure for immunoblotting was previously described [72]. Briefly, proteins were loaded into the lanes of a sodium dodecyl sulfate polyacrylamide gel (SDS-PAGE) or tricine sodium dodecyl sulfate polyacrylamide gel (Tricine-SDS-PAGE). The proteins were separated by electrophoresis and transferred to nitrocellulose membranes (0.45 or 0.22 μm, Schleicher & Schuell). The membranes were blocked with 5% nonfat dry milk in 0.01 M PBS (pH 7.4) and 0.05% Tween-20 (TPBS) at room temperature for 1 hour. Subsequently, the membrane was incubated with primary antibodies directed against target proteins overnight at 4°C. The final dilutions for primary antibodies were: 6E10 (1:1,000), LC3 (1:2,000), and Beclin1 (1:2,000). After three quick washes in TPBS, the membranes were incubated with secondary antibodies conjugated to horseradish peroxidase (Amersham) diluted at 1:5,000 in TPBS for 1 hour. The immuno-complexes were detected by the enhanced chemiluminescence method (Amersham, RPN2106). The density of immunoblotting was quantified with the software of Quantity One (Bio-Rad Laboratories).

### Assaying Aβ_42_ degradation

The analysis for Aβ_42_ degradation in the NG2 cell line was performed as previously described [24]. NG2 cell line (5 × 10^5^cells/well) cultured in Sato medium containing 1% horse serum was exposed to human Aβ_42_ (400 nM). Aβ_42_ levels in cell-culture supernatants and adherent cells were determined by immunoblotting. For some experiments, NG2 cell line was incubated with HiLyte Fluor™ 488-Aβ_42_ (400 nM) for 6 hours, followed by the treatment of leupeptin (20 μM), pepstatin A (20 μM), wortmannin, and bafilomycin A1(0.2 nM) for 18 hours. The protein levels of Aβ_42_, LC3, and beclin1 were determined by immunoblotting.

### Real-time PCR

NG2 cell line was incubated with HiLyte Fluor™ 488-labeled Aβ_42_ (400 nM) for the indicated times. Total RNA extraction and reverse transcription for NG2 cell line and cerebral cortex were performed as previously reported [72] with some modifications. Briefly, total RNA was extracted using Trizol reagent (Invitrogen, 15596018). After treated with RNase-free DNase I (Roche Applied Science, 10104159001), first strand cDNA was synthesized with M-MLV reverse transcriptase (Promega, M1701) and Oligo-dTs (Promega, C1101). Real-time quantitative PCR was conducted with ABI Prism 7500 Sequence Detection System according to the instruction of the manufacturer (Applied Biosystems). Dissociation curve analyses were performed using the instrument’s default setting immediately after each PCR run to ensure specificity. The expression level of target genes was normalized to the actin gene. The primers which were used for real time PCR are provided in the following Table 1.

**Table 1:** Primers for real time PCR

**Gene**	**Primer**
Mouse-*LAMP1*-sense	CAGCACTCTTTGAGGTGAAAAAC
Mouse-*LAMP1*-antisense	ACGATCTGAGAACCATTCGCA
Mouse-*LAMP2*-sense	ATATGTGCAACAAAGAGCAGGT
Mouse-*LAMP2*-antisense	TGCCAATTAGGTAAGCAATCACT
Mouse-*NG2*-sense	CCCCCCCATACCCATGTC
Mouse-*NG2*-antisense	CGATCGGAAATAACCTGAAGCT
Mouse-*Acti*n-sense	CAACGAGCGGTTCCGAT
Mouse-*Actin*-antisense	GCCACAGGATTCCATACCCA

### Small RNA interference

Small interfering RNAs were synthesized by Shanghai GenePharma Co., Ltd. The siRNA sequences for mouse *beclin1*, siRNA-1: sense 5′-GAGGAGCCAUUUAUUGAAACUCG-3′ and antisense 5′-CGAGUUUCAAUAAAUGGCUCCUC-3′; siRNA-2: sense 5′-GGACAACAAGUUUGACCAUGC-3′ and antisense 5′-GCAUGGUCAAACUUGUUGUCC-3′; for siRNA control, oligos with no matching GeneBank sequence were used: sense 5′-GCGACGAUCUGCCUAAGAU-3′ and antisense 5′-AUCUUAGGCAGAUCGUCGC-3′. The pairs of siRNA oligonucleotides were prepared as a 20 μM stock. For transient transfection, NG2 cell line was cultured in six-well plates to 80% confluence and transfected with Lipofectamine™ RNAiMAX (Invitrogen, 13778–150) according to the manufacturer’s instructions. After transfection, the cells were left for another 24 hours before they were used for experiments.

### Quantification of the mCherry-LC3 puncta

Human microtubule-associated protein 1 light chain 3 beta (MAP1LC3B, Gene ID: 81631) was cloned from a cDNA of HEK-293 (ATCC, CRL-1573) with forward primer (5′- CAA CAA GCT TCC ATG CCG TCG GAG AAG ACC -3′) and reverse primer (5′- CGC GGA TCC TTA CAC TGA CAA TTT CAT CCC G -3′). The nucleotide sequence was inserted into mCherry-C1 vector (a gift from Dr. Kang, JS, INS, SIBS, CAS) at the Hind III and BamH I restriction sites. The plasmid was verified by sequencing (Invitrogen). The mCherry–LC3 was transiently transfected NG2 cell line using Lipofectamine™ 2000 (Invitrogen, 11668–019) following the manufacturer’s instructions. After transfection, the cells were treated with HiLyte Fluor™ 488-labeled Aβ_42_ (400 nM) for 18 hours. After that, cells were fixed in 4% paraformaldehyde, permeabilized with 0.1% Triton X-100, and incubated with DAPI (1 μg/ml) for 5 minutes. Cells were examined using the Olympus BX51 microscope system equipped with a DP72 digital camera (Olympus America Inc.).

To analyze the formation number of autophagosomes, we determined mCherry–LC3 (the plasmid was kindly provided by Dr. Jiansheng Kang) puncta in cells. The cells were classified as (a) cells with diffuse mCherry–LC3 fluorescence or with few mCherry–LC3 puncta (<20 dots/cell) and (b) cells with numerous mCherry–LC3 puncta (>20 dots/cell), representing autophagosomes [73]. At least 200 cells per sample were scored for each condition in three independent experiments. The percentage of mCherry-LC3–positive cells with mCherry-LC3 punctate dots were calculated [74].

### Statistical analysis

Statistical analysis was assessed by ANOVA followed by Student–Newman–Keuls analyses. An unpaired *t* test was used for the analysis of quantitative data of bafilomycin A1. Two-way ANOVA was used for the analysis of quantitative data of activated NG2 cells number. Data were presented as means ± SD. Difference in which p < 0.05 was considered statistically significant.

## Abbreviations

AD: Alzheimer’s disease; ALP: Autophagy/lysosome pathway; ApoE: Apolipoprotein E; APP: Amyloid β precursor protein; APPsw: Swedish mutant APP; ATPase: Adenosine triphosphatase; Aβ: Amyloid β peptide; Baf: Bafilomycin A1; bFGF: Fibroblast growth factor basic; BSA: Bovine serum albumin; CNS: Central nervous system; CytoD: Cytochalasin D; DAPI: 4′,6-diamidino-2-phenylindole; DMEM: Dulbecco’s modified eagle medium; FPRL1: Formyl peptide receptor-like 1; GAPDH: Glyceraldehyde-3-phosphate dehydrogenase; GTPase: Guanosine triphosphatase; LAMP1: Lysosome-associated membrane glycoprotein 1; LAMP2: Lysosome-associated membrane glycoprotein 2; LC3B: Microtubule-associated protein 1 light chain 3 beta; LDLR: Low-density lipoprotein receptor; Leu: Leupeptin; LIR: LC3-interacting region; MAG: Myelin-associated glycoprotein; mRNA: Messenger RNA; NCDZ: Nocodazole; NFTs: Neurofibrillary tangles; OPC: Oligodendroglial precursor cells; PAS: Pre-autophagosomal structure; PBS: Phosphate buffer saline; PDGF AA: Platelet-derived growth factor-AA; PE: Phophatidylethanolamine; Pep: Pepstatin A; PI3K: Phosphatidylinositol 3-kinases; RAGE: Scavenger receptor for advanced glycation end products; Rpm: Revolutions per minute; RT-PCR: Real time–polymerase chain reaction; siRNA: Small interfering RNAs; Thio-s: Thioflavine S; TLRs: Toll-like receptors; UPP: Ubiquitin/proteasome pathway.

## Competing interests

The authors declare that they have no competing interests.

## Authors’ contributions

WL, YT, ZF, YM and GY carried out all of the experiments. ZK and JL participated in the design of the study and the writing of the manuscript. All authors read and approved the final manuscript.
